# Juvenile idiopathic scoliosis treated with posterior arthrodesis and segmental pedicle screw instrumentation before the age of 9 years: a 5-year follow-up

**DOI:** 10.1186/1748-7161-4-1

**Published:** 2009-01-06

**Authors:** Ahmet Yılmaz Şarlak, Halil Atmaca, Levent Buluç, Bilgehan Tosun, Resul Musaoğlu

**Affiliations:** 1 Kocaeli University, School of Medicine, Department of Orthopaedics and Traumatology, Umuttepe Merkez Kampüsü, 41380 Umuttepe, Kocaelı, Turkey

## Abstract

**Study design:**

Retrospective study.

**Objective:**

To evaluate the radiological results of fusion with segmental pedicle screw fixation in juvenile idiopathic scoliosis with a minimum 5-year follow-up.

**Summary of background data:**

Progression of spinal deformity after posterior instrumentation and fusion in immature patients has been reported by several authors. Segmental pedicle screw fixation has been shown to be effective in controlling both coronal and sagittal plane deformities. However, there is no long term study of fusion with segmental pedicle screw fixation in these group of patients.

**Methods:**

Seven patients with juvenile idiopathic scoliosis treated by segmental pedicle screw fixation and fusion were analyzed. The average age of the patients was 7.4 years (range 5–9 years) at the time of the operation. All the patients were followed up 5 years or more (range 5–8 years) and were all Risser V at the most recent follow up. Three dimensional reconstruction of the radiographs was obtained and 3DStudio Max software was used for combining, evaluating and modifying the technical data derived from both 2d and 3d scan data.

**Results:**

The preoperative thoracic curve of 56 ± 15° was corrected to 24 ± 17° (57% correction) at the latest follow-up. The lumbar curve of 43 ± 14° was corrected to 23 ± 6° (46% correction) at the latest follow-up. The preoperative thoracic kyphosis of 37 ± 13° and the lumbar lordosis of 33 ± 13° were changed to 27 ± 13° and 42 ± 21°, respectively at the latest follow-up. None of the patients showed coronal decompensation at the latest follow-up. Four patients had no evidence of crankshaft phenomenon. In two patients slight increase in Cobb angle at the instrumented segments with a significant increase in AVR suggesting crankshaft phenomenon was seen. One patient had a curve increase in both instrumented and non instrumented segments due to incorrect strategy.

**Conclusion:**

In juvenile idiopathic curves of Risser 0 patients with open triradiate cartilages, routine combined anterior fusion to prevent crankshaft may not be warranted by posterior segmental pedicle screw instrumentation.

## Background

Children diagnosed with scoliosis after 3 years and before 10 years constitute 8–21 % of those with scoliosis and present a distinct clinical entity. Approximately 70% of curves in patients with juvenile idiopathic scoliosis progress and require surgery.

Deformity progression after posterior fusion for idiopathic scoliosis have been addressed across all Risser groups but Risser 0 patients with open triradiate cartilage are at most risk for crankshaft [1,2]. The incidence of crankshaft phenomenon in Risser 0 patients with open triradiate cartilage treated with segmental pedicle screw instrumentation – posterior fusion followed till maturity; have not been reported in English speaking literature to our knowledge.

The purpose of this paper is to analyze 7 idiopathic juvenile cases with scoliosis followed to maturity with respect to the results of segmental pedicle screw instrumentation and posterior fusion.

## Methods

The records of 120 consecutive patients who underwent posterior segmental instrumentation and fusion with the diagnosis of idiopathic scoliosis from 1995 through 2008 were reviewed. The criteria for inclusion in the current study were: a) a diagnosis of juvenile idiopathic scoliosis, b) posterior segmental instrumentation and fusion with pedicle screws, c) Risser sign of 0 at the time of operation, d) Risser sign of 5 at latest follow-up assessment. In all, seven patients with a diagnosis of juvenile idiopathic scoliosis corrected by pedicle screw instrumented fusions and followed up for a minimum of five years were retrospectively analyzed with respect to the results of the surgical procedure. All patients had primary and secondary curves. According to the Lenke classification, 2 patients were type 1, 4 type 3 and 1 type 5. Considering the curve magnitude brace treatment was not attempted preoperatively in any of the patients.

The mean patient age at the time of surgical procedure was 7.4 years (range, 5–9 years) and the male/female ratio was 1:6. All girls were premenarchal preoperatively. All patients were observed up 5 years or more (range, 5–8 years) and were all Risser V at most recent follow-up (Additional file 1).

### Radiographic evaluation

**Figure 1:**
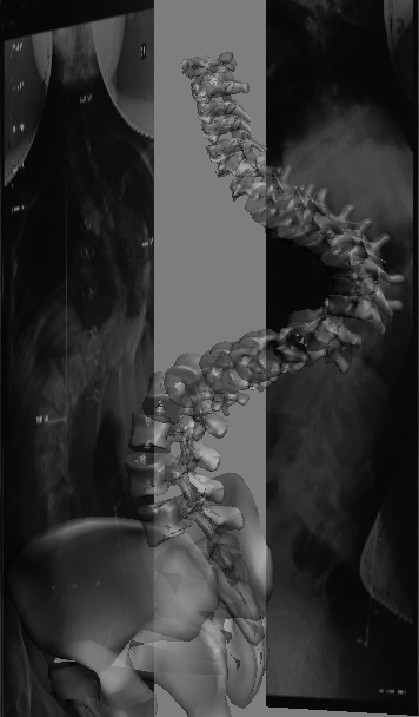
**Anteroposterior and lateral 2d scan data was mapped on the background of front and left/right view of 3Dsmax as reference images**.

Preoperative standing long-cassette anteroposterior (AP) and lateral radiographs, as well as right and left bending coronal radiographs were reviewed. Standing long-cassette AP and lateral radiographs from three different time periods (preoperative, immediate postoperative [1–6 weeks], and latest follow-up) were evaluated to determine deformity correction and changes in radiographic characteristics over time. The radiograms were scanned to a computer workstation by using a transparent media scanner (Mikrotek^® ^MRS-3200A3). Scanned images were digitized using the Silverfast Ai6 Professional Scan Software. To obtain a 3D model of the human spine, a solid spine and pelvis model was scanned using a 3D scanner (Next Engine Desktop 3D Scanner Model 2020, USA). The same scanner was used to obtain a 3D virtual model of the spine and pelvis. 3DStudio Max software was used for combining, evaluating and modifying the technical data derived from both 2d and 3d scan data. Frontal and lateral 2d scan data was mapped on the background of front and left/right view of 3Dsmax as reference images (Figure 1). To calibrate the system for the 3D reconstruction, the scales on the radiographs were used. Sagittal corpus height was measured on the lateral radiographs. Then, with the calibration of the virtual model's vertebral heights; the model was equalized to the real spine. The virtual model was set to be transparent in order to visualize the radiographs lying on the back font.

**Figure 2:**
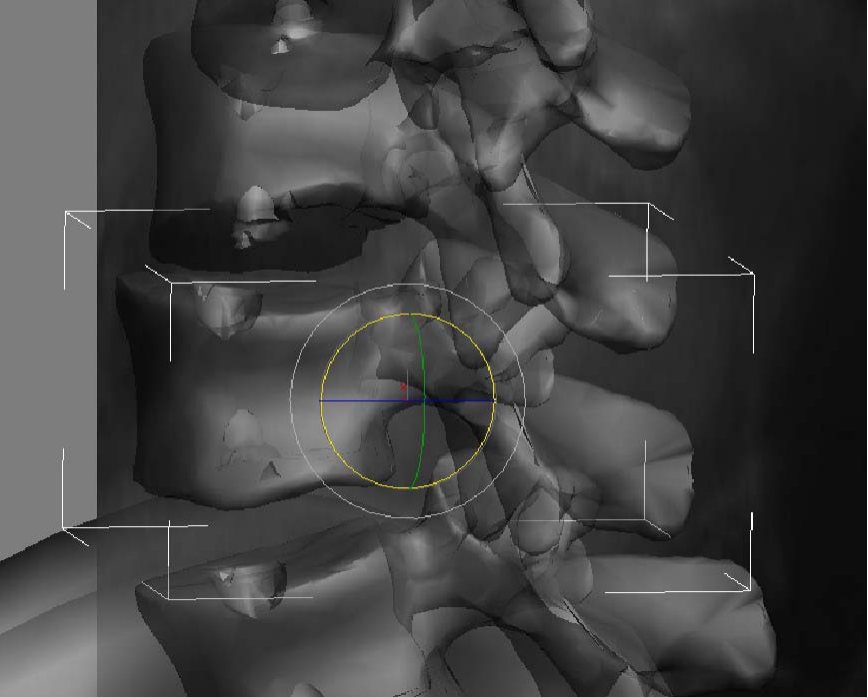
**The 3D virtual model can be manipulated in three coordinates (coronal, sagittal and axial)**.

Each vertebral segment can be manipulated on the x, y, and z coordinates using the 3DsMax software (Figure 2). Starting from the L5 vertebra and proceeding to the cephalad segments, each vertebral segment was placed on the AP radiograph. The borders of the pedicle shadows, spinous process, the superior, inferior end plates and the lateral borders of the vertebral corpus were used for the matching of the model to the radiograph. In the frontal plane (AP radiograph); y (tilt) and z (rotation) coordinates were manipulated. In the sagittal plane, using the same principle, the vertebral segments were only manipulated in the x coordinate. 3DsMax software is capable of determining the angular value changes of the scoliotic model using the x, y, and z coordinates to the reference angular value: x represents the sagittal plane measurements (kyphosis and lordosis), y represents the coronal plane measurements (tilt), and z represents the axial plane measurements (rotation) (Figure 2).

A digital programme (Canvas 9.0) was used for analyzing the measurements. The deformity was measured on all radiographs by the Cobb method using the end vertebrae determined on the preoperative standing radiographs. An approximation of skeletal maturity was assessed according to the Risser scale from the preoperative AP radiograph.

The stable vertebra was defined as the vertebra most nearly bisected by the central sacral vertical line (CSVL). Additional criteria measured from the AP radiographs were thoracic Cobb angle, lumbar Cobb angle, apical vertebral translation (AVT). AVT for the thoracic curve was measured relative to the coronal C7 plumbline, and the lumbar curve apical vertebra was measured relative to the CSVL.

Global coronal balance was determined by measuring the horizontal distance between the C7 plumbline and CSVL. By convention, shift of the C7 plumbline to the left is considered negative balance, while a shift to the right is considered positive. A trunk shift more than 2 cm was considered postoperative decompensation.

Sagittal analysis at each of the radiographic examination period was performed using the lateral radiographs. Global sagittal balance was determined by measuring the horizontal distance from a vertical line extended from the center of the C7 vertebral body relative to the posterior superior cortex of the body of the sacrum. By convention, C7 plumbline falling in front of the posterior S1 endplate represent positive sagittal balance, while those falling behind the endplate represent a negative balance. Regional sagittal alignment was determined from T5 to T12.

### Surgical technique

**Figure 3:**
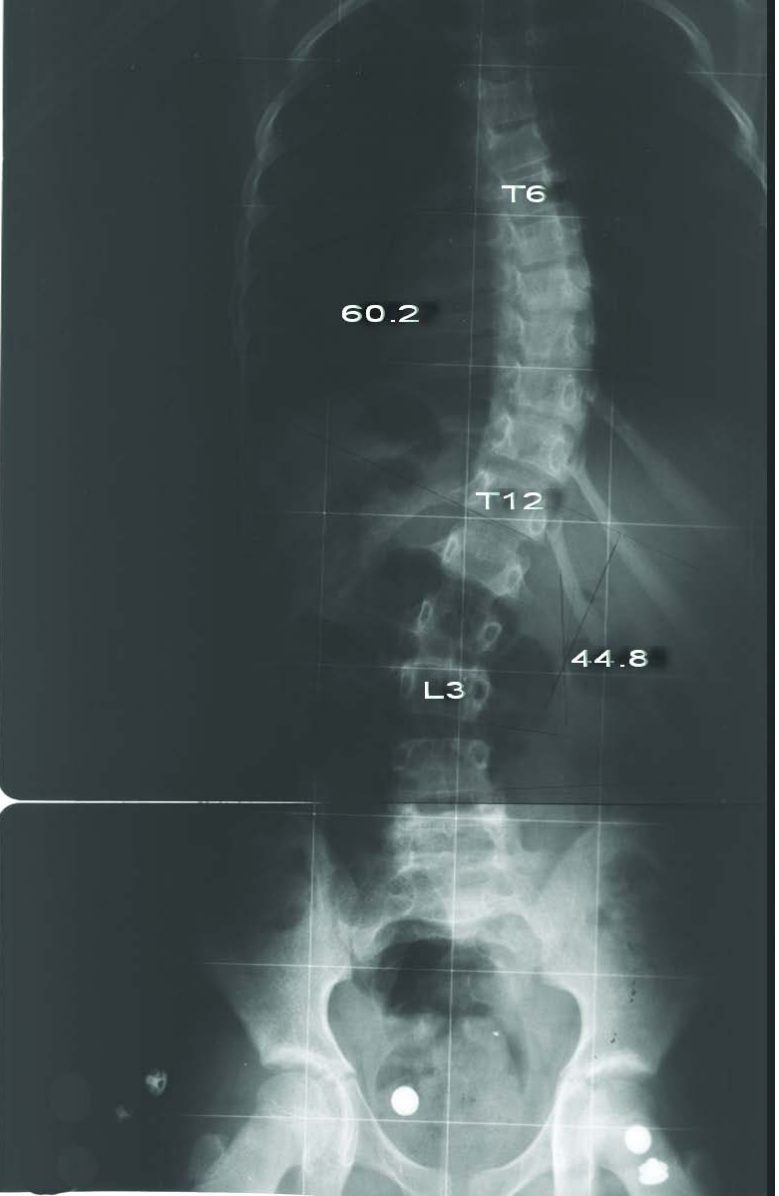
**Eight-year-old girl with Lenke type 3BN scoliosis (Patient no 1)**. Preoperative anteroposterior radiograph shows 60.2° main thoracic curve and 44.8° lumbar curve.

**Figure 4:**
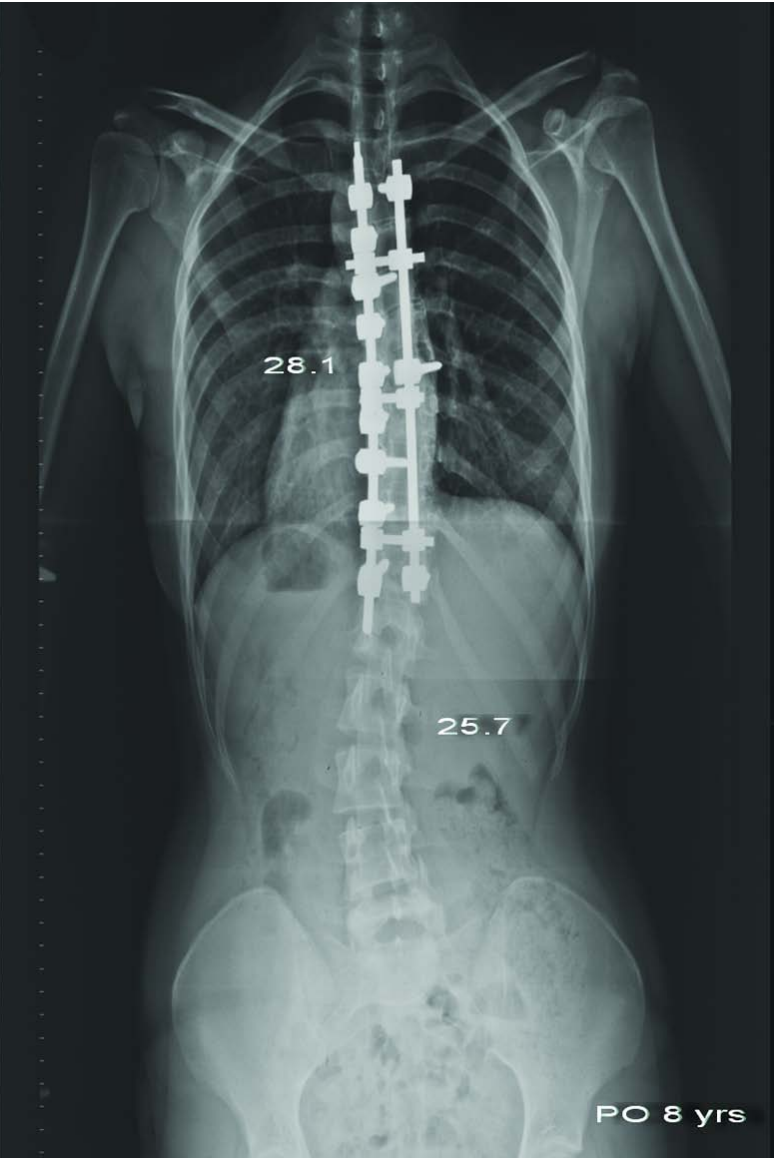
**Anteroposterior radiograph of the same patient taken 8 years after surgery**. The main thoracic curve was 28.1° and the lumbar curve was 25.7°. Coronal alignment was well maintained.

**Figure 5:**
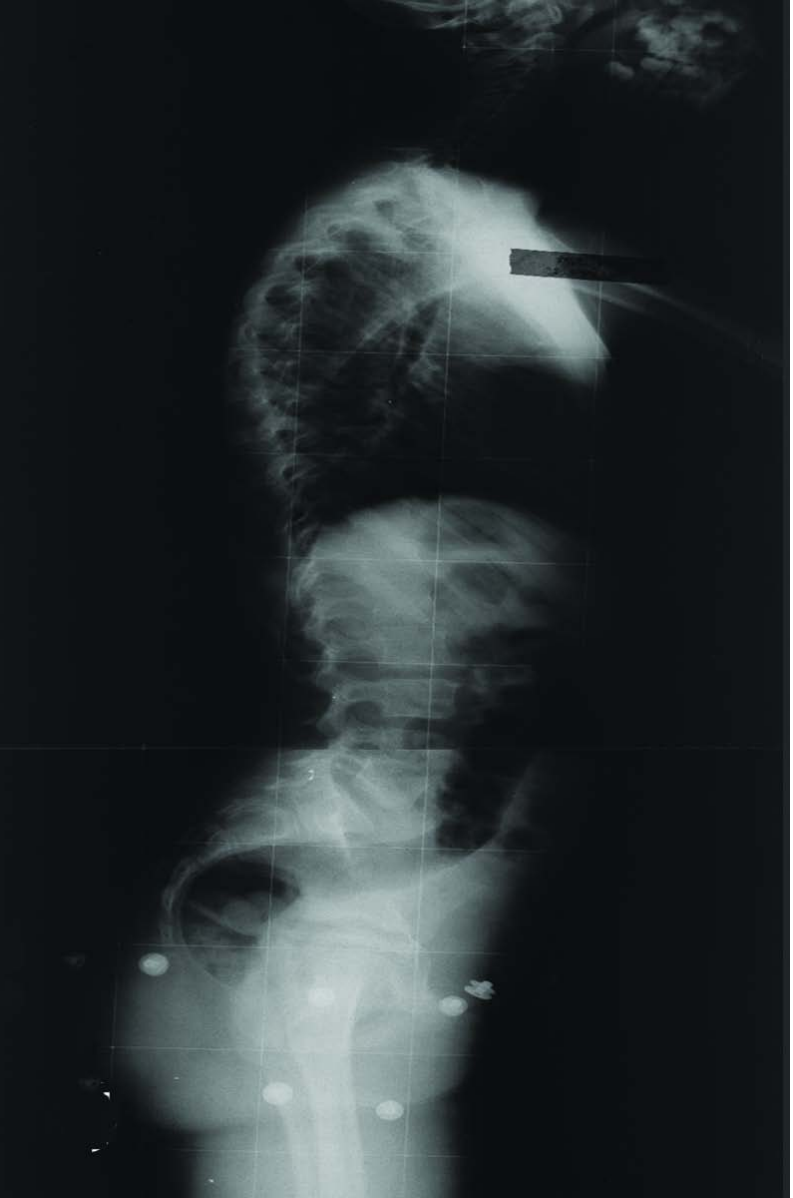
**Preoperative lateral radiograph of the same patient**.

**Figure 6:**
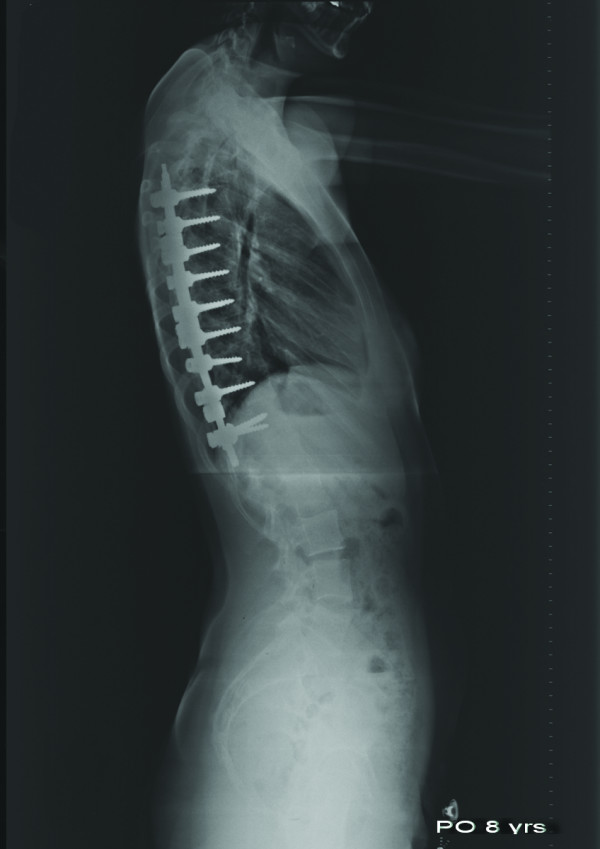
**Postoperative lateral radiograph of the same patient taken 8 years after surgery**. Sagittal alignment was well maintained.

All surgeries were carried out by the senior author (AYS) and the pedicle screws were inserted using the technique described by Suk [3]. The fusion levels were determined according to the flexibility of the structural curve on bending radiographs. Fusion was usually carried out from upper neutral to lower neutral vertebrae. Insertion was done segmentally on the concave side of the thoracic curve and every other or third vertebra on the convex side. Following the screw insertion, rigid rod contouring to normal sagittal curve of the instrumented spinal segments was inserted on the concave side. Correction of the curve was achieved solely by rod derotation. After locking the concave rod in the corrected position, convex side rod contouring was done and locked in situ. In most cases, three transverse connectors were used to enhance the rigidity of the instrumentation. Following instrumentation, the posterior fusion was performed using cancellous chips (Figure 3, 4, 5 and 6).

All the patients were out of bed and began ambulation on the second postoperative day. No patients were braced after surgery.

Statistical analysis was performed using SPSS (version 13.0; Chicago, IL). Friedman test was used to compare the parameters in terms of preoperative, early postoperative and latest follow-up periods. Significance was defined as p < 0.05.

## Results

### Deformity correction

#### Coronal curve correction

In the coronal plane, the preoperative thoracic curve of 56 ± 15° (range, 28–81) was corrected to 24 ± 14° (range, 7–46) at the most recent follow-up showing a correction of 57% (range, 43–83%). Lumbar curve of 43 ± 14° (range, 21–60) was corrected to 23 ± 6° (range, 9–30) at the most recent follow-up, showing a correction rate of 46% (range, 0–78%). The differences were significant both in thoracic and lumbar curves among the three time periods (p = 0,001 and p = 0,012, respectively) (Additional file 2). In four of our patients without crankshaft phenomenon slight increase of distal vertebral tilt, without an increase in both vertebral rotation- Cobb angle was seen.

#### Sagittal curve correction

Preoperative thoracic kyphosis of 37 ± 13° (range, 15–53) was changed to 27 ± 13° (range, 10–51) at the most recent follow-up, showing a significant difference (p = 0.05). Lumbar lordosis of 33 ± 13° (range, 6–45) was changed 42 ± 21° (range, 3–63) at the most recent follow-up, showing no significant changes during the follow-up (p = 0.27) (Additional file 3).

#### Axial curve correction

In the axial plane, the preoperative thoracic AVR of 15,9 ± 3.9° (range, 11–20) was corrected to 6,7 ± 2,2° (range, 4–10) postoperatively, showing a correction of 57%. At the most recent follow-up the thoracic AVR was 9,2 ± 4° (range, 7–46). In two patients a significant increase in AVR suggesting crankshaft phenomenon was seen (patients 4 and 6). The preoperative lumbar AVR of 12,6 ± 3,7° (range, 10–20) was corrected to 5,1 ± 2,2° (range, 2–8) postoperatively, showing a correction of 58%. At the most recent follow-up the lumbar AVR was 5,7 ± 2,7° (range, 0–9), showing an insignificant correction loss. (Additional file 4).

### Spinal balance

#### Coronal balance

Before surgery, 4 of 7 patients were imbalanced > 20 mm (31, 21, 13, and 21 mm). Of 4 patients decompensated before surgery, 2 patients showed decompensation, postoperatively (38 and 40 mm). At the most recent follow-up, none of the patients showed decompensation (Additional file 2).

#### Sagittal balance

Changes in the global sagittal balance observed after surgery were usually transient. After surgery, GSB shifted an average of 3.8 mm forward to before surgery with an average of 1 mm. At the most recent follow-up, however, the average GSB returned to 16.6 mm (Additional file 3).

### Apical vertebrae translation

Preoperative AVT for the thoracic curve of 31.6 ± 12.8 mm (range, 10–48) was improved to 14.3 ± 10.7 mm (range, 5–33) postoperatively and was 14.4 ± 8.8 mm (range, 2.6–24.7) at the most recent follow-up. There were no significant differences among the groups. Preoperative AVT for the lumbar curve of 30.5 ± 22 mm (range, 0–65.9) was improved to 15.6 ± 8.7 mm (range, 3–27) postoperatively and was 21.1 ± 10.5 mm (range, 3–32) at the most recent follow-up showing no significant difference (Additional file 2).

One superficial wound infection (patient 2) healed uneventfully with debridement and delayed closure. While screw misplacement was suspected on postoperative plain radiography in some of our patients, there were no neurologic, vascular or visceral complications related to the screw malposition.

At latest follow-up, the average body height of the patients was 150.8 cm (range, 137–168 cm). All of the measurements were between the third and 97^th ^percentile according to the growing charts. Dysmorphy or disproportional trunk/limbs length due to spinal longitudinal growth arrest were not observed in any of our patients.

## Discussion

Surgical treatment is not as clearly indicated for juvenile idiopathic scoliosis as its for adolescent idiopathic scoliosis. There are two main options available: fusion and non-fusion techniques when surgery being considered in a young child.

Non-fusion techniques have been suggested to correct progressive deformity while preserving longitudinal spinal growth. Age at initial instrumentation, the number of instrumented spinal segments, intrinsic spinal growth will individually affect any gain in spinal length [4]. However, currently neither the effect of spinal fusion on the normal loss of vital capacity with aging nor the minimum length of thoracic spine needed at the time of skeletal maturity for adequate thoracic volume and capacity is known [5]. Dissatisfaction with outcomes, high rate of complications with the non-fusion techniques has led to search for improved methods of surgical technique. Dual posterior growing rod construct [6-8], Shilla technique [9], growth modulation procedures have recently became popular [10].

Correction loss, crankshaft phenomenon, implantation failure and lack of spinal growth had been the main concerns for the traditional posterior instrumentation and fusion in immature patients with scoliosis [2,11-13].

Crankshaft phenomenon had been defined as deformity progression after posterior fusion consistent with anterior growth – vertebral rotation in the fused segments [14]. A progression of Cobb angle ≥ 10°, rib vertebrae angle difference ≥ 10° ; AVR by Perdriolle method to be ≥ 5° were accepted as the usual criteria for the diagnosis of crankshaft phenomenon [2,12,13,15,16]. Dubousset pointed out that curve progression occurs in the face of thick fusion masses as well as rigid instrumentation [14]. Therefore, in the scoliotic deformities of children ≤ 10 years and who have Risser 0 sign with open triradiate cartilages, anterior growth arrest was recommended in addition to posterior fusion[1,2]. There has been a trend towards the use of thoracic pedicle screws in deformity surgery based on the clinical advantages in terms of greater rigidity and improved fusion rates when compared with other forms of fixation [17,18]. Ability to resist crankshaft phenomenon using multilevel thoracic pedicle screws as reported by Suk is a convenient way of avoiding anterior procedures in at risk skeletally immature patients [3].

Consistent with the findings of Suk in our small series of seven patients with juvenile idiopathic scoliosis; in five patients segmental pedicle screws were effective to prevent crankshaft phenomenon. In two patients slight increase in Cobb angle at the implanted segments with an increase in AVR suggesting crankshaft phenomenon was seen (patients 4 and 6). The problem with the measure of rotation had been the limited accuracy of plane radiographic technique, as well as difficulties in assessing rotational correction when radiographic landmarks are obscured by instrumentation after surgery. Though it's a time consuming procedure, this inaccuracy has been tried to be overcome by the 3D reconstruction in our study [19] (Additional file 5). Consistent with anterior growth further reduction of kyphosis and a slight increase of lumbar lordosis was seen in most of our patients. This may well suggest that, pedicle screws may slow anterior growth but the main affect may be the greater control of rotation preventing crankshaft in the majority of cases. Postoperative coronal decompensation was not detected in any of our patients. Negative sagittal imbalance is known to be better tolerated than positive sagittal imbalance [20]. Our study has shown the potential for subsequent negative sagittal imbalance though it may well be due to standard lateral radiographic positioning with the arms forward flexed [21].

**Figure 7:**
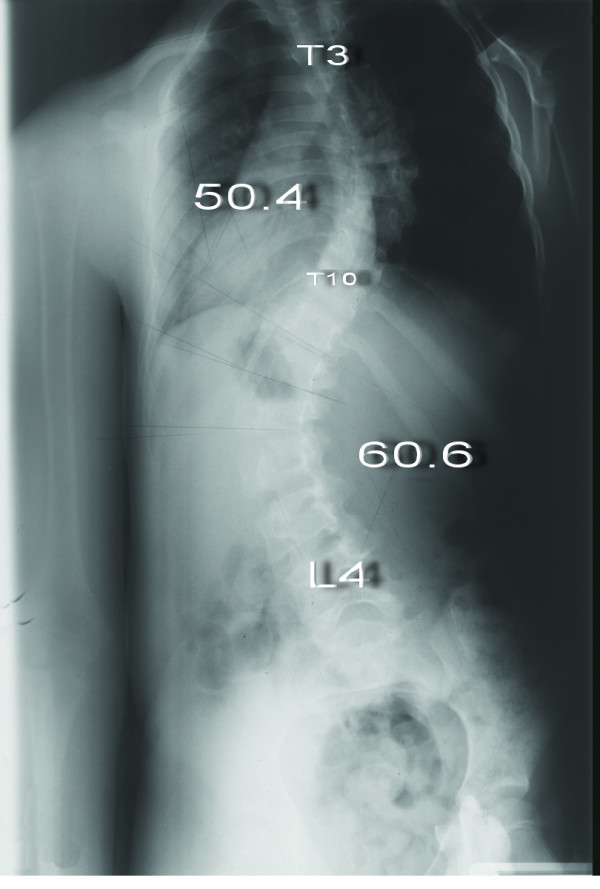
**Eight-year-old girl with Lenke type 3CN scoliosis (Patient no 7) Preoperative anteroposterior radiograph shows 50.4° thoracic curve and 60.6° lumbar curve**.

**Figure 8:**
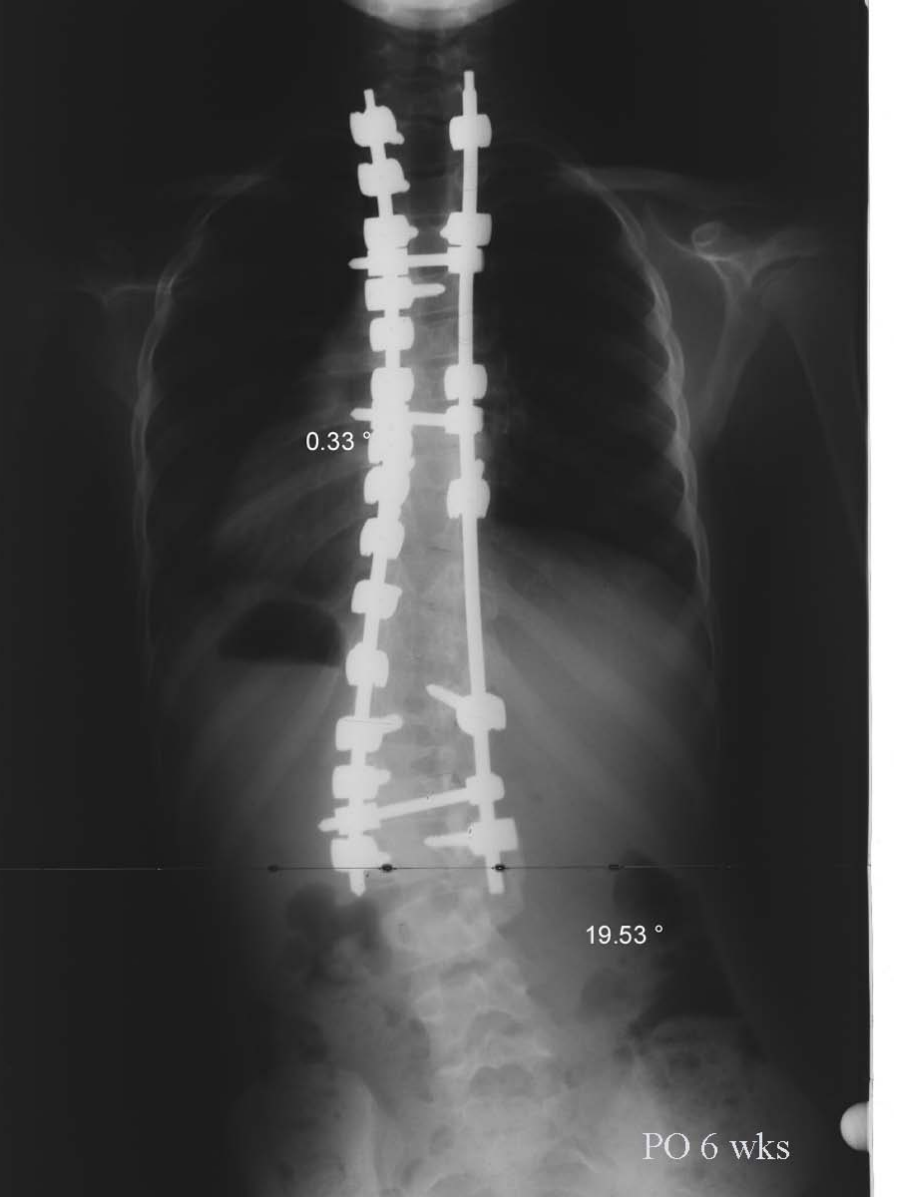
**Anteroposterior radiograph of the same patient taken 6 weeks after surgery**. The main thoracic curve was corrected to 0.3° and the lumbar curve was corrected to 19.5°.

**Figure 9:**
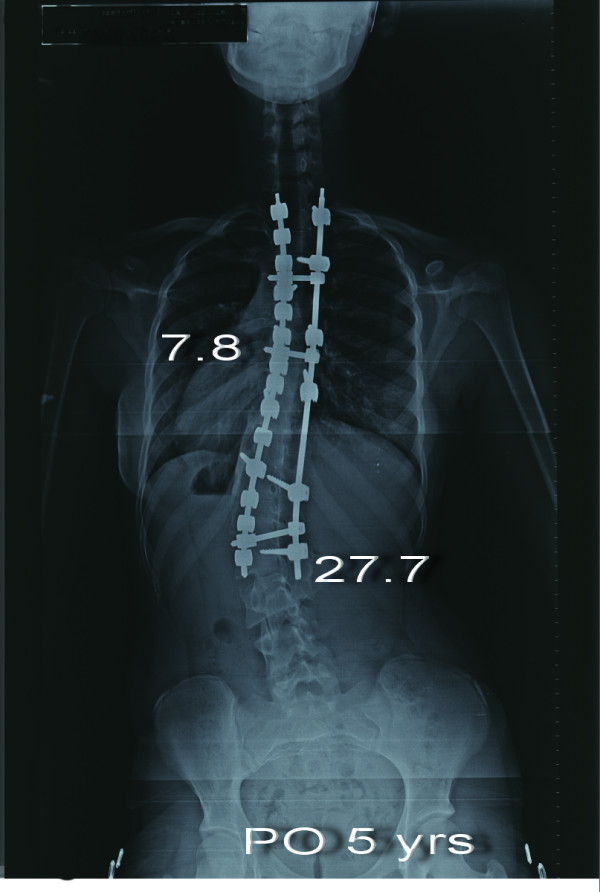
**Anteroposterior radiograph of the patient taken 5 years after surgery**. The thoracic curve was 7.8° and the lumbar curve was 27.7°. Increase of vertebral tilt in distal segments is notable.

**Figure 10:**
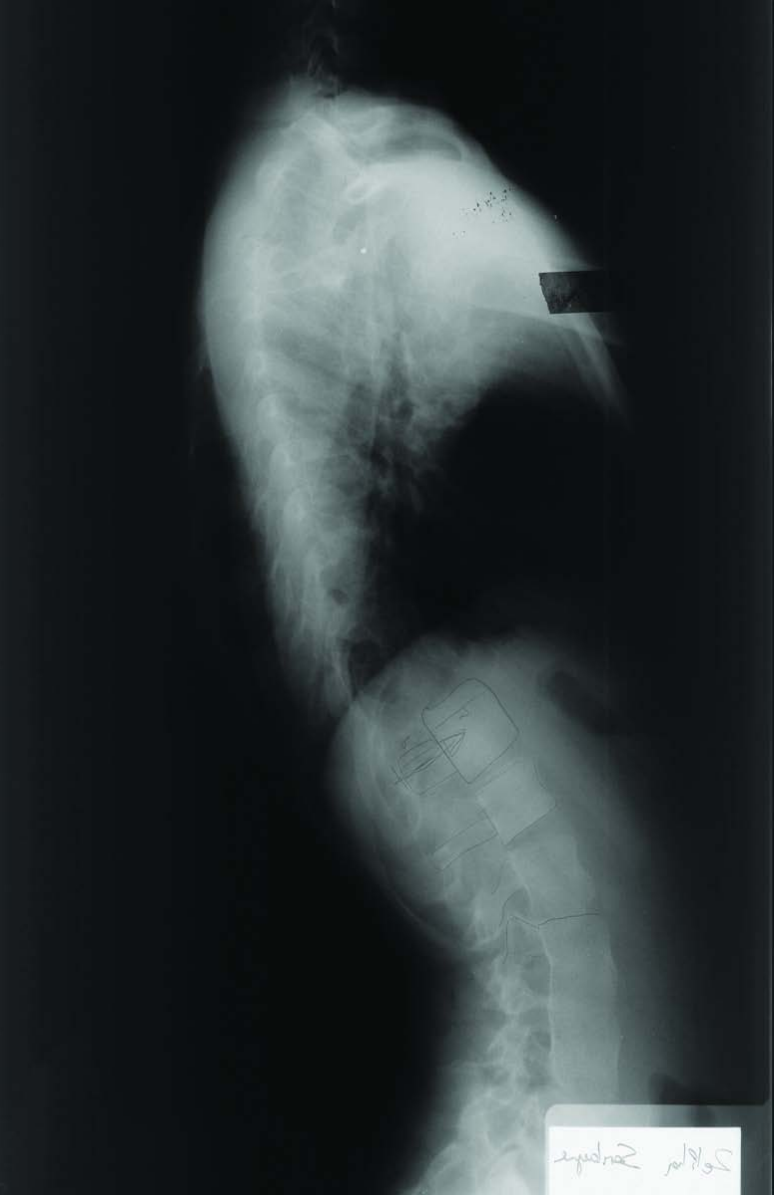
**Preoperative lateral radiograph of the patient**.

**Figure 11:**
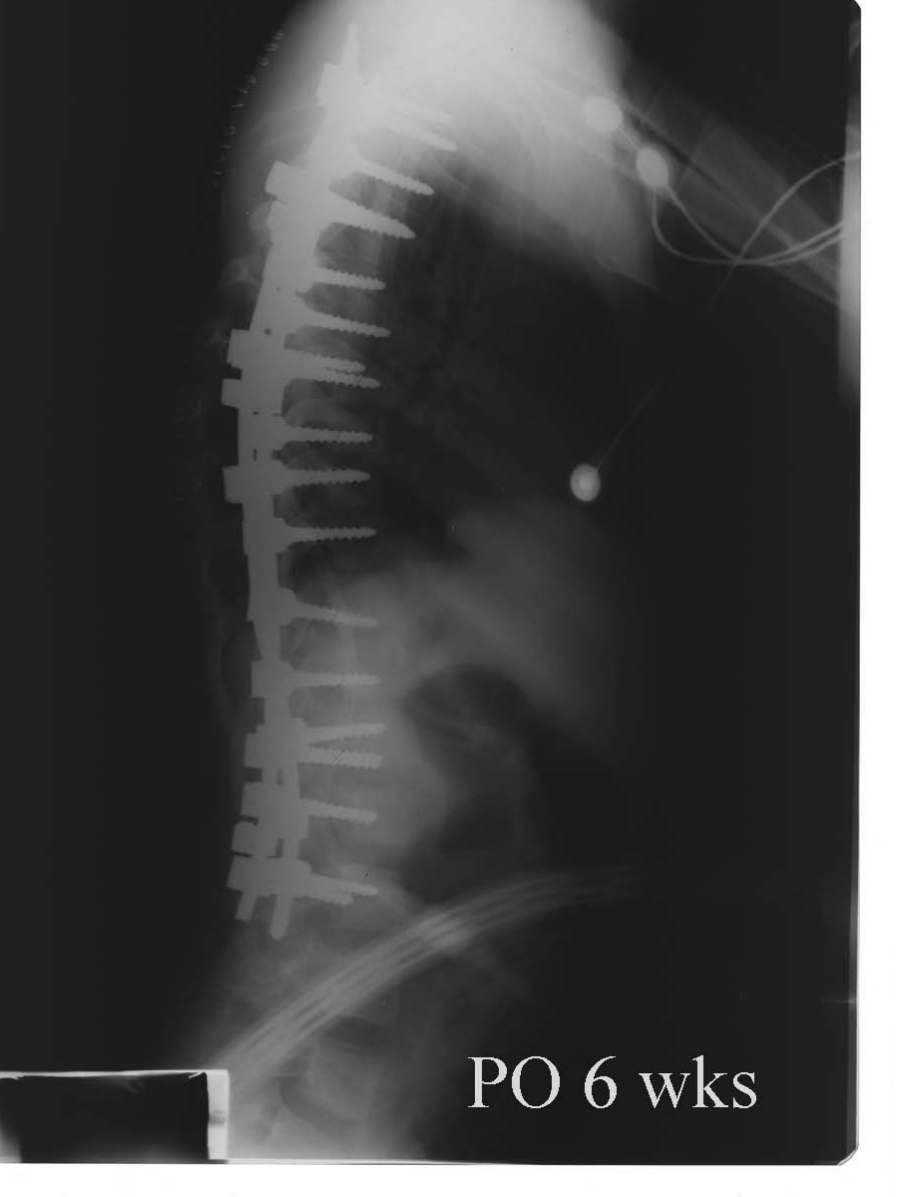
**Postoperative lateral radiograph of the same patient taken 6 weeks after surgery**. Thoracic kyphosis and lumbar lordosis angles were 44.9° and 35.5°, respectively.

**Figure 12:**
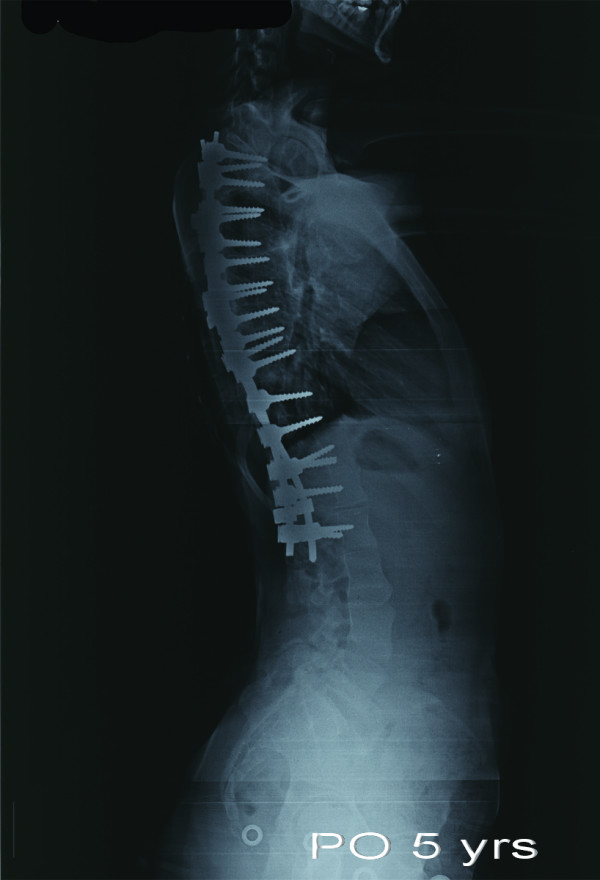
**Postoperative lateral radiograph taken 5 years after surgery**. Thoracic kyphosis angle was reduced to 10.1°.

Derotation maneuver [22-25], over correction [26-28], improper fusion level [24,25], have been shown to have significant impact on the behavior of noninstrumented segments. One of our patients is a good example of incorrect strategy. Over correction and inappropriate proximal and distal fusion level in a Lenke type 3C curve caused curve increase in both instrumented and noninstrumented segments with a significant increase of vertebral tilt in distal segments (Figure 7, 8, 9,10, 11 and 12) (Additional file 6).

Studies of three dimensional vertebral body rotation, translation, and angulation have demonstrated that there is a direct, nonlinear relationship between the fused and unfused vertebral segments [29,30]. In four of our patients without crankshaft phenomenon slight increase of distal vertebral tilt, without an increase in both vertebral rotation- Cobb angle is a matter of concern. The increase in vertebral tilt in the nonfused segments of our patients cannot be defined as "spinning out" as it was described to be loss of correction with increased rotation below the instrumented segments [22,26,31]. We agree with Asher that compensatory curves do not have any further capacity to compensate in the transverse plane [32].

Our study suggest that; routine combined anterior fusion to prevent crankshaft may not be warranted by posterior segmental pedicle instrumented fusions of juvenile idiopathic curves in the selected group of Risser 0 patients with open triradiate cartilages. A larger patient population in patients treated in a similar fashion to substitute our findings is necessary.

## Competing interests

The authors declare that they have no competing interests.

## Authors' contributions

AYS conceived of the study and participated in the design of the study, HA performed the statistical analysis, LB and BT participated in its design and coordination of the study, RM participated in the sequence alignment. All authors read and approved the final manuscript.

## Supplementary Material

Additional file 1 **Clinical and radiographic data of the patients.** The data provided represent the demographics of the patients. Click here for file

Additional File 2 **Coronal deformity correction.** The data provided represent the statistical analysis of the coronal plane deformity. Click here for file

Additional File 3 **Sagittal deformity correction.** The data provided represent the statistical analysis of the sagittal plane deformity. Click here for file

Additional File 4 **Axial deformity correction.** The data provided represent the statistical analysis of the axial plane deformity. Click here for file

Additional File 5 **3-D measurement results of patient 1 with satisfactory result.** The data provided represent the 3-D measurement results of patient 1. Click here for file

Additional File 6 **3-D measurement results of patient 7 with unsatisfactory result.** The data provided represent the 3-D measurement results of patient 7. Click here for file
