# Supplementary material for: Juvenile idiopathic scoliosis treated with posterior arthrodesis and segmental pedicle screw instrumentation before the age of 9 years: a 5-year follow-up
Source: Scoliosis. 2009 Jan 6;4:1. doi: 10.1186/1748-7161-4-1 (PMC2633314; doi:10.1186/1748-7161-4-1)
Supplement: Additional file 1 — Clinical and radiographic data of the patients. The data provided represent the demographics of the patients. [file 1748-7161-4-1-S1.doc]

| **Patient no** | **Age at operation (yrs)** | **Follow-up (yrs)** | **Lenke** | **Thoracic Cobb Preop(°)** | **Thoracic Cobb Latest(°)** | **Lumbar Cobb**  **Preop(°)** | **Lumbar Cobb**  **Latest(°)** | **Treatment** |
| --- | --- | --- | --- | --- | --- | --- | --- | --- |
| 1 | 8 | 8 | 3BN | 60.2 | 28.1 | 44.8 | 25.7 | PSF T4-T12 |
| 2 | 9 | 7 | 5CN | 28.8 | 9.3 | 43.1 | 21.1 | PSF T5-L3 |
| 3 | 9 | 5 | 3CN | 81.1 | 46.3 | 55.2 | 30.6 | PSF T3-L4 |
| 4 | 7 | 6 | 1CN | 66.1 | 36.7 | 49.4 | 25.6 | PSF T5-L2 |
| 5 | 6 | 5 | 3BN | 51.5 | 14.5 | 43.8 | 9.5 | PSF T3-L3 |
| 6 | 5 | 7 | 1AN | 55.9 | 25.7 | 26.9 | 22.9 | PSF T4-L1 |
| 7 | 8 | 5 | 3CN | 50.4 | 7.8 | 60.6 | 27.7 | PSF T1-L2 |

**Table 1.** Clinical and radiographic data of the patients. PSF= posterior spinal fusion.
