# Supplementary material for: Juvenile idiopathic scoliosis treated with posterior arthrodesis and segmental pedicle screw instrumentation before the age of 9 years: a 5-year follow-up
Source: Scoliosis. 2009 Jan 6;4:1. doi: 10.1186/1748-7161-4-1 (PMC2633314; doi:10.1186/1748-7161-4-1)
Supplement: Additional File 2 — Coronal deformity correction. The data provided represent the statistical analysis of the coronal plane deformity. [file 1748-7161-4-1-S2.doc]

| **Patient** | **Thoracic curve** | | | **Lumbar curve** | | | **AVT(mm)** | | | | | | GCB(mm) | | |
| --- | --- | --- | --- | --- | --- | --- | --- | --- | --- | --- | --- | --- | --- | --- | --- |
|  | Preop | Postop  6 weeks (corr) | Latest  (corr) | Preop | Postop  6 weeks (corr) | Latest (corr) | Preop | | Postop  6 weeks | | Latest | | Preop | Postop  6 weeks | **Latest** |
|  |  |  |  |  |  |  | T | L | T | L | T | L |  |  |  |
| 1 | 60.2° | 22.7° | 28.1° | 44.8° | 23.5° | 25.7° | 28.9 | 13.1 | 5.00 | 22.6 | 11.1 | 10.0 | 0 | -12.6 | **-14.1** |
| 2 | 28.8° | 5. 2° | 9. 3° | 43. 0° | 28.6° | 21.1° | 10.8 | 65.9 | 5.70 | 12.4 | 6.30 | 32.0 | -31.4 | -13.6 | **-17.7** |
| 3 | 81.1° | 43.2° | 46.3° | 55.2° | 26.9° | 30.6° | 36.9 | 59.5 | 24.0 | 27.1 | 22.2 | 27.2 | 58.4 | 38.2 | **12.2** |
| 4 | 66.1° | 32.1° | 36.7° | 49.4° | 21.1° | 25.6° | 44.1 | 21.8 | 33.2 | 12.5 | 23.3 | 22.2 | 0 | -5. 4 | **-8. 1** |
| 5 | 51.5° | 11.3° | 14.5° | 43.8° | 4. 1° | 9. 5° | 48.7 | 20.3 | 9.80 | 3.11 | 11.2 | 24.8 | -21.8 | -40.2 | **-10.1** |
| 6 | 55.9° | 20.1° | 25.7° | 26.9° | 11.3° | 22.9° | 23.2 | 0.00 | 16.10 | 8.67 | 24.7 | 3.24 | -13.6 | -15.1 | **0** |
| 7 | 50.4° | 0. 3° | 7. 8° | 60.6° | 19.5° | 27.7° | 29.4 | 33.2 | 6.51 | 23.2 | 2.60 | 28.4 | -21.4 | -18.1 | **-6.2** |
| **Average** | **56±15°** | **19±15° (65%)** | **24±17°**  **(57%)** | **43±14°** | **19±8°**  **(55%)** | **23±6°**  **(46%)** | 31.69  ±12.84 | 30.54  ±22.03 | 14.33  ±10.77 | 15.65  ±8.78 | 14.49 ±8.87 | 21.12  ±10.54 | 20.97 | 20.48 | **9.77** |
| p value | 0.001 | | | 0,012 | | |  |  |  |  |  |  |  |  |  |

**Table 2.** Coronal deformity correction. AVT= apical vertebra translation; GCB= global coronal balance. Significant values (p<0.05) are marked with bold font.
