# Supplementary material for: Juvenile idiopathic scoliosis treated with posterior arthrodesis and segmental pedicle screw instrumentation before the age of 9 years: a 5-year follow-up
Source: Scoliosis. 2009 Jan 6;4:1. doi: 10.1186/1748-7161-4-1 (PMC2633314; doi:10.1186/1748-7161-4-1)
Supplement: Additional File 3 — Sagittal deformity correction. The data provided represent the statistical analysis of the sagittal plane deformity. [file 1748-7161-4-1-S3.doc]

| **Patient** | **Thoracic kyphosis** | | | **Lumbar lordosis** | | | GSB(mm) | | |
| --- | --- | --- | --- | --- | --- | --- | --- | --- | --- |
|  | Preop | Postop  6 weeks | Latest | Preop | Postop  6 weeks | Latest | Preop | Postop  6 weeks | **Latest** |
|  |  |  |  |  |  |  |  |  |  |
| 1 | 51.8° | 20.4° | 18.7° | 49.2° | 56.5° | 56.3° | 18.5 | 9.1 | **-39.6** |
| 2 | 26.9° | 19.1° | 17.5° | 43.4° | 26.1° | 28.4° | -1.5 | 0.1 | **0.1** |
| 3 | 43.1° | 30.3° | 28.3° | 35.8° | 26.3° | 3.3° | -13.1 | -17.2 | **-31.7** |
| 4 | 38.4° | 36.2° | 34.1° | 6.9° | 32.3° | 37.9° | 9.1 | -9.6 | **-26.3** |
| 5 | 32.1° | 35.3° | 28.7° | 39.2° | 50.7° | 63.3° | 10.1 | 8.8 | **67.4** |
| 6 | 15.8° | 35.3° | 51.5° | 31.3° | 34.2° | 61.6° | 24.3 | 6.6 | **-39.9** |
| 7 | 53.4° | 44.9° | 10.1° | 25.7° | 35.5° | 44.9° | -40.1 | 28.7 | **-46.3** |
| **Average** | **37±13°** | **31±9°** | **27±13°** | 33±13° | 37±11° | 42±21° | 16.7 | 11.4 | **35.9** |
| p value | 0.05 | | | **0.27** | | |  |  |  |

**Table 3.** Sagittal deformity. GSB= global sagittal balance. Significant values (p<0.05) are marked with bold font.
