# Supplementary material for: Juvenile idiopathic scoliosis treated with posterior arthrodesis and segmental pedicle screw instrumentation before the age of 9 years: a 5-year follow-up
Source: Scoliosis. 2009 Jan 6;4:1. doi: 10.1186/1748-7161-4-1 (PMC2633314; doi:10.1186/1748-7161-4-1)
Supplement: Additional File 4 — Axial deformity correction. The data provided represent the statistical analysis of the axial plane deformity. [file 1748-7161-4-1-S4.doc]

| **Patient** | **Thoracic AVR** | | | **Lumbar AVR** | | |
| --- | --- | --- | --- | --- | --- | --- |
|  | preoperative | early post operative | latest follow-up | preoperative | early post operative | latest follow-up |
| 1 | 19,46 | 10,20 | 11,09 | 9,55 | 4,10 | 5,80 |
| 2 |  |  |  | 20,50 |  | 0,87 |
| 3 | 11,02 | 7,00 | 8,36 | 12,84 | 3,24 | 4,73 |
| 4 | **20,03** | **6,39** | **11,00** | 13,10 | 2,70 | 4,71 |
| 5 | 18,97 | 4,09 | 4,55 | 10,16 | 5,80 | 6,46 |
| 6 | **13,42** | **8,14** | **15,11** | 10,28 | 8,12 | 9,51 |
| 7 | 12,78 | 4,66 | 5,18 | 11,76 | 7,19 | 8,03 |
| Average | 15,95±3,97 | 6,75±2,26 | 9,22±4,01 | 12,60±3,74 | 5,19±2,20 | 5,73±2,76 |
|  |  |  |  |  |  |  |

**Table 4.** Axial deformity correction. AVR= Apical Vertebra Rotation. An increase in thoracic AVR in patients 4 and 6 suggesting crankshaft phenomenon was seen. (Bold font).
